# Effects of various types of organo-mica on the physical properties of polyimide nanocomposites

**DOI:** 10.1038/s41598-023-51064-6

**Published:** 2024-01-05

**Authors:** Hara Jeon, Changyub Na, Lee Ku Kwac, Hong Gun Kim, Jin-Hae Chang

**Affiliations:** 1 Graduate School of Carbon Convergence Engineering, Jeonju University, Jeonju, 55069 Korea; 2 Institute of Carbon Technology, Jeonju University, Jeonju, 55069 Korea

**Keywords:** Engineering, Materials science, Nanoscience and technology

## Abstract

Poly(amic acid) (PAA) was synthesized using dianhydride 4,4’-oxydiphthalic anhydride and diamine 3,3'-dihydroxybenzidine, and polyimide (PI) hybrid films were synthesized by dispersing organo-mica in PAA through a solution intercalation method. Hexadimethrine-mica (HM-Mica), 1,2-dimethylhexadecylimidazolium-mica (MI-Mica), and didodecyldiphenylammonium-mica (DP-Mica), which were obtained via the organic modification of pristine mica, were used as the organo-micas for the PI hybrid films. The organo-mica content was varied from 0.5 to 3.0 wt% with respect to the PI matrix. The thermomechanical properties, morphology, and optical transparency of the resultant PI hybrid films were measured and compared. Dispersion of even small amounts of organo-mica effectively improved the physical properties of the PI hybrids, and maximum enhancements in physical properties were observed at a specific critical content. Electron microscopy of the hybrid films revealed that the organo-mica uniformly dispersed throughout the polymer matrix at the nanoscale level when added at low contents but aggregated in the matrix when added at levels above the critical content. Structural changes in the organo-mica closely influenced the changes in the physical properties of the hybrid films. All PI hybrid films with various organo-mica contents showed similar optical properties, but that prepared with MI-Mica demonstrated the best thermomechanical properties. All synthesized PI hybrid films were transparent regardless of the type and content of organo-mica used.

## Introduction

Aromatic polyimide (PI) is a polymer with a low-crystallinity or amorphous structure featuring transparent and rigid chains. Owing to these characteristics, PI has excellent heat and chemical resistance, mechanical and electrical properties, and dimensional stability. Thus, it is widely used in automobiles, aerospace applications, and electrical and electronic materials^1–3^. PI is also known to have physical properties suitable for application to flexible display substrates, and research on this topic is actively being conducted^4^.

Because PI is a high-performance material that can be used under extreme conditions given its excellent various thermomechanical properties, synthesizing PI with excellent physicochemical properties is possible by modifying the monomers used for PI and optimizing the reaction conditions. Further research on hybrid materials in which a filler characterized with excellent dispersibility and excellent interfacial adhesion properties is uniformly dispersed at the nanoscale level in the PI matrix could be conducted to obtain new super-engineered PI materials with excellent physical properties that cannot be developed using conventional manufacturing processes.

PI composites obtained from a blend of straight, rigid, and flexible structures and novel nanosized fillers have been extensively studied to improve their physical properties and expand their application scope^5,6^. However, obtaining nanoscale composites by mixing different structures with rigid or flexible properties is challenging because two substances with different properties rarely dissolve in the same solvent simultaneously; in most cases, phase separation occurs. When synthesizing PI with excellent optical properties, the main chain must have a bent structure or an asymmetric substituent. In general, PIs that provide excellent optical properties have poor thermomechanical properties. Thus, a nanocomposite design that partially uses monomers with rigid structures that do not reduce the overall thermomechanical properties of PI while maintaining its optical transparency must be developed to compensate for this disadvantage^7,8^.

Polymer nanocomposites obtained by uniformly dispersing clay in a nano-sized polymer matrix show significantly improved properties, such as excellent thermal stability and mechanical properties, a high heat deflection temperature, and good dimensional resistance, compared with those of conventional materials obtained by simply mixing composite materials^9–11^. Previous research has confirmed that clay could be well dispersed in the matrices of polyamides, polyesters, and PIs at the nanoscale level. Clay, which is mainly used for nanocomposites, is characterized with thermal stability, stiffness, and barrier properties owing to the orientation of plate-like clay sheets. Therefore, dispersing an appropriate amount of clay in a polymer matrix can greatly improve significantly the physical properties of the hybrid^12–14^. Commonly used smectite-based clays are classified into various types such as saponite, hectorite, bentonite, montmorillonite, and mica. Even a small amount of clay with a large surface area (700–800 m^2^/g) can considerably enhance the physical properties of the hybrid material via the mutual attraction between the clay and polymer matrix. In addition, when clay with an aspect ratio (length/width, L/W) of approximately 200–2,000 is completely dispersed in the polymer matrix, the heat resistance, thermomechanical properties, solvent resistance, and insulation properties of the hybrid films are enhanced^13,15^.

Mica with a high aspect ratio (L/W = 1230) has excellent compatibility and can induce excellent thermomechanical tensile properties while maintaining the original optical properties of the hybrid material if dispersed at the nanoscale level in the polymer matrix. Therefore, among various smectite-based clays utilized as fillers for nanocomposite materials, mica is known as a filler with the greatest ability to enhance physical properties. In general, hydrophilic mica has poor miscibility or compatibility with lipophilic polymers^16,17^. Organo-mica synthesized through the organic modification of pristine mica can be evenly dispersed at the nanometer scale in a polymer matrix. However, to the best of our knowledge, the applicability of different types of organically modified mica to the preparation of PI hybrid films has not been attempted^18–20^.

The purpose of this study is to synthesize a new PI using a diamine monomer with a hydroxyl group and to manufacture a PI hybrid film by synthesizing three new types of organo-mica that can be dispersed in nanoscale in hydrophilic PI. In addition, the thermomechanical properties, clay dispersibility, and optical transparency of hybrid films prepared with various types and contents of organo-mica were investigated, and the properties were compared.

In this study, dianhydride 4,4’-oxydiphthalic anhydride (ODPA) and diamine 3,3'-dihydroxybenzidine (DHB) monomers were used to synthesize PI. The use of DHB, which contains –OH groups, facilitated the dispersion of the polymer chain through hydrogen bonding with the hydrophilic clay layer. Thus, excellent physical properties can be expected from the hybrid film even in the presence of a small amount of clay. Three new types of organo-micas were synthesized, and PI hybrid films with various filler contents were prepared. The organo-micas hexadimethrine-mica (HM-Mica), 1,2-dimethylhexadecylimidazolium-mica (MI-Mica), and didodecyldiphenylammonium-mica (DP-Mica) were obtained through the substitution reactions of pristine Na^+^-type fluorinated synthetic mica (Na^+^-mica) with the organic materials hexadimethrine bromide (HM), 1,2-dimethylhexadecylimidazolium chloride (MI), and didodecyl-diphenylammonium chloride (DP), respectively. The morphology of the clay dispersed in the PI matrix was observed using a transmission electron microscope (TEM) to explain the change various physical properties of the hybrid films according to the clay content.

## Methods

### Materials

The ODPA and DHB used to synthesize PI in this experiment were purchased from TCI (Tokyo, Japan). The HM (MW = 4,000–6,000) used to prepare HM-Mica was purchased from Aldrich (Yongin, Korea), and both MI and DP were purchased from Duksan Chem. (Suwon, Korea). Na^+^-Mica was supplied by CO-OP Ltd. (Tokyo, Japan). The cation-exchange capacity of the mica is 70–80 meq/100 g. *N,N'*-Dimethylacetamide (DMAc) was purchased from Aldrich (Yongin, Korea) and used as a solvent. The moisture components present in the solvent were completely removed using a 4 Å molecular sieve.

### Preparation of organo-mica

**Scheme 1:**
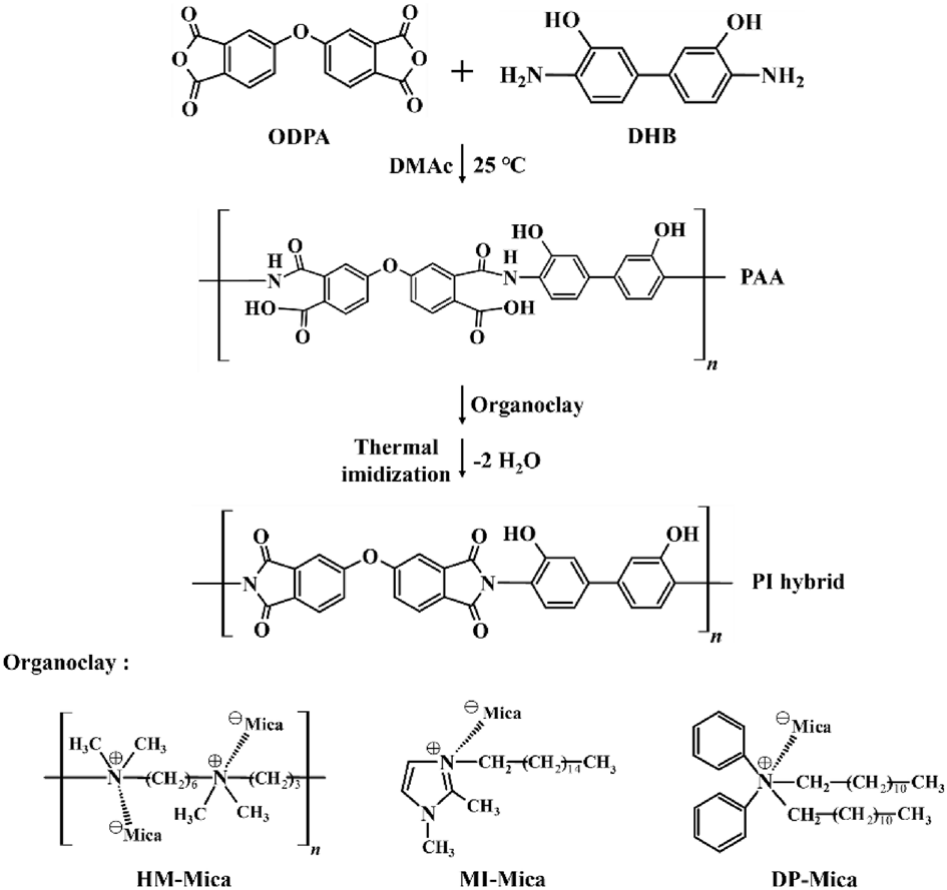
Synthesis route for the fabrication of the PI hybrid films.

The organo-micas used in this study were synthesized using an ion-exchange reaction between pristine Na^+^-Mica and various organic materials. HM-Mica, MI-Mica, and DP-Mica were synthesized via a multistep pathway. Because the synthesis methods of all three types of organo-mica are similar, only the synthesis of HM-Mica is described here. A total of 6.55 g (1.75 × 10^−2^ mol) of HM-Br^-^ was dissolved in 100 mL of deionized water and heated to 70 °C for 2 h. A dispersion of 20.0 g of Na^+^-Mica in deionized water (200 mL) was added to the HM-Br^-^ solution, and the mixture was vigorously stirred at 70 °C for 5 h. The precipitate obtained by filtration was dispersed in a mixed solvent of 300 mL of water and ethanol (v/v = 50/50) and stirred for 2 h. The product was filtered and freeze-dried to obtain a white powder. The chemical structures of the organo-micas are shown in Scheme 1.

### Synthesis of PAA and the PI hybrid films

Because all synthesis methods used to obtain the PI hybrid films with various filler types and contents are nearly the same, only the method for preparing a PI hybrid film containing 1 wt% HM-Mica is described here. PAA was synthesized by dissolving ODPA (3.10 g; 1.00 × 10^−2^ mol) and DHB (2.16 g; 1.00 × 10^−2^ mol) in 40 mL of DMAc and reacting at room temperature for 14 h. The inherent viscosity of PAA was measured at a concentration of 0.2 g/dL solution in DMAc at 25 °C and the value was 1.01. The PAA (5.26 g; 1.00 × 10^−2^ mol) solution was stabilized in a vacuum maintained first at 50 °C and then at 80 °C for 1 h each time. Dispersion was improved by mixing 0.05 g of HM-Mica in 20 mL of DMAc solution and sonicating for 3 h. This solution was mixed with PAA, followed by three cycles of 30 min of stirring and 30 min of ultrasonication. The mixed solution was cast on a glass plate and heat-treated in an oven for various temperatures and times in a vacuum. Details of the heat-treatment conditions for the PI hybrid films are summarized in Table 1. The thickness of the hybrid PI films obtained under various heat treatments remained constant at 19–22 μm regardless of the type and content of organo-mica used. The PI hybrid synthesis process is shown in Scheme 1. An attempt was made to synthesize a PI hybrid film containing more than 3 wt% of organo-mica, but bubbles were generated during the polymerization process and the film was broken into several pieces. From these results, it is believed that excessive mica content hinders the formation of PI hybrid films.

**Table 1:** Heat-treatment conditions for the PI hybrid films.

Samples	Temperature (°C)/Time (h)/Pressure (Torr)
PAA	25/14/760 → 50/1/1 → 80/1/1
PI hybrid	110/0.5/1 → 140/0.5/1 → 170/0.5/1 → 195/1/1 → 220/1/1 → 235/2/1

### Characterization

Various characterization techniques were employed to analyze the properties of the PI and PI hybrid films. Fourier transform-infrared (FT-IR) spectroscopy was performed using a Perkin Elmer L-300 (London, UK) instrument to identify the functional groups present in the PI structure. The spectroscopic analysis was conducted in the wavenumber range of 4000–1000 cm^-1^. The resolution and scan speed of FT-IR were 32 cm^-1^ and 0.2 cm/s, respectively. Solid-state ^13^C cross-polarized/magic angle spinning nuclear magnetic resonance (NMR) spectroscopy was performed using a Bruker 400 DSX (Berlin Germany) instrument with a Larmor frequency of 100.61 MHz to verify the structure of PI.

Wide-angle X-ray diffraction (XRD) analysis was performed using a Rigaku D/Max-IIIB X-ray diffractometer (Tokyo, Japan) with Ni-filtered Cu-Kα radiation. The scan rate for the XRD measurements was 2°/min in the 2θ range of 3°–15°. The morphology of the clay dispersed in the PI matrix was investigated using a JEOL JEM 2100 TEM (Tokyo, Jpan) operated at an acceleration voltage of 120 kV. Samples for TEM analysis were prepared by curing the films on epoxy resin and cutting the resin into 90-nm-thick specimens with a microtome under vacuum conditions. The observations were conducted at various magnifications.

Differential scanning calorimetry (DSC) and thermogravimetric analysis (TGA) were used simultaneously to measure the thermal properties of the PI hybrid films. A Netzsch F3200 differential scanning calorimeter (Munchen, Germany) and a TA Q-500 thermogravimetric analyzer (New Castle, USA) were employed, and the temperature was changed at a rate of 20 °C/min under a N_2_ atmosphere. The inflow amount of nitrogen was 30 ml/min. Thermomechanical analysis (TMA) was performed using a Seiko TMA/SS100 (Tokyo, Japan) instrument to measure the coefficient of thermal expansion (CTE) of the PI hybrid films. The temperature was increased at a rate of 5 °C/min under a load of 0.1 N. In order to obtain reliable results, the CTE was determined by performing the first heating at 50 to 200 °C and then the second heating at the same temperature condition.

The mechanical tensile properties of the PI hybrid films were determined using a Shimadzu JP/AG-50KNX (Tokyo, Japan) universal tensile machine. The measurements were conducted at a crosshead speed of 5 mm/min, and the average values were obtained after excluding measurements outside the error range. The experimental errors of the ultimate tensile strength and initial modulus were within ± 1 MPa and ± 0.05 GPa, respectively. The light transmittance (λ_o_ and 500 nm^trans^) and yellowness index (YI) of the PI hybrid films were measured using a Shimadzu UV-3600 UV–vis spectrometer (Tokyo Japan) and a Konica Minolta CM-3600d spectrophotometer (Tokyo, Japan), respectively.

## Experimental results

### FT-IR and ^13^C-NMR analyses

**Figure 1:**
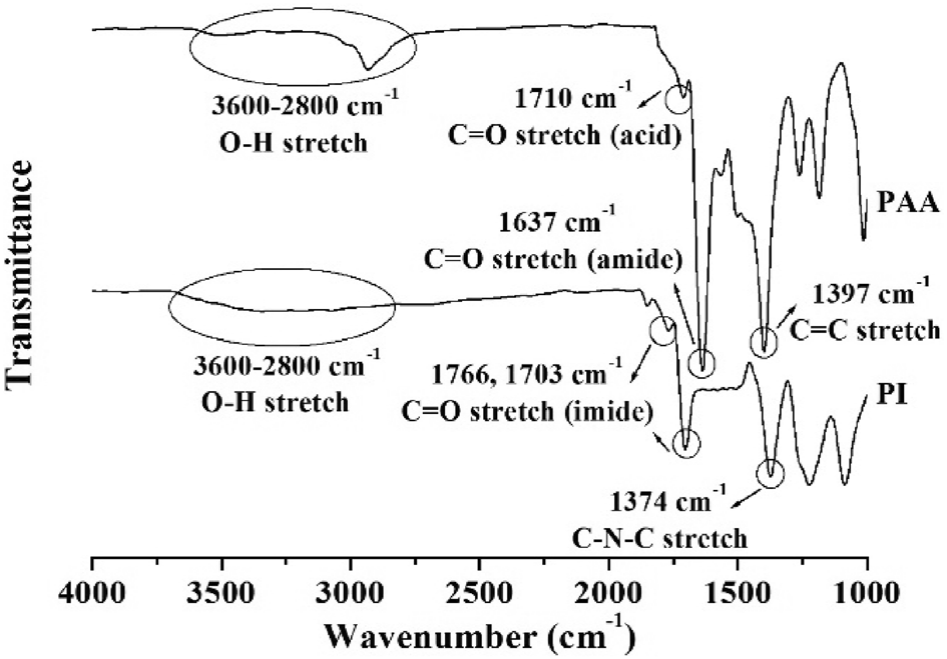
FT-IR spectra of PAA and PI.

The Fig. 1 shows the functional groups in the PI structure. In the FT-IR spectrum of PAA, a very broad –OH band was observed between 2800 and 3600 cm^−1^ and a C = O stretching band was observed between 1710 (acid) and 1637 (amide) cm^−1^. A C = C stretching band was also observed at 1397 cm^−1^. In the FT-IR spectrum of PI, C = O stretching bands were observed at 1780 and 1698 cm^-1^ and a characteristic band indicating the imide functional group was observed at 1370 cm^−1^. This band corresponds to the stretching vibrations of the C–N–C bond in imides. The presence of imide functional groups suggests the occurrence of a reaction involving a carboxylic and an amide groups, resulting in the formation of the imide^21^.

**Figure 2:**
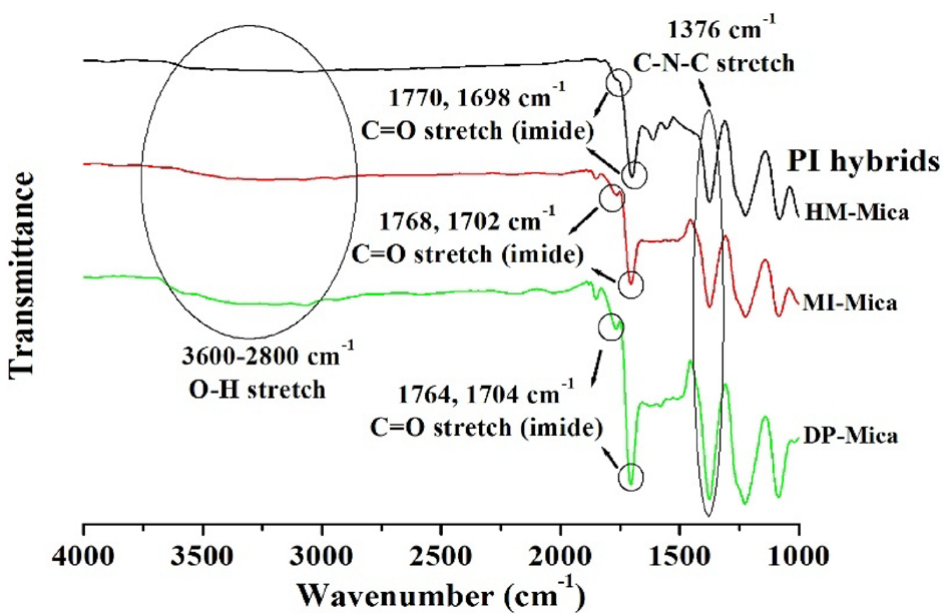
FT-IR spectra of PI hybrid films containing 3 wt% organo-mica content.

The FT-IR spectra of all PI hybrids containing 3 wt% of the three types of organo-mica are shown in Fig. 2. All hybrids contain PI with substituted –OH groups and organo-mica composed of various alkyl groups. The –OH group contained in the PI main chain was observed in a wide range of 3600–2800 cm^−1^. Unfortunately, the aromatic alkyl group (–CH_2_–) appearing between 3200 and 3000 cm^-1^ and the aliphatic alkyl group appearing between 3000 and 2800 cm^-1^ could not be identified because the bands overlapped with the -OH group visible in a wide area. Additionally, clays dispersed in the hybrid may interfere with detailed observation of the bands of functional groups.

**Figure 3:**
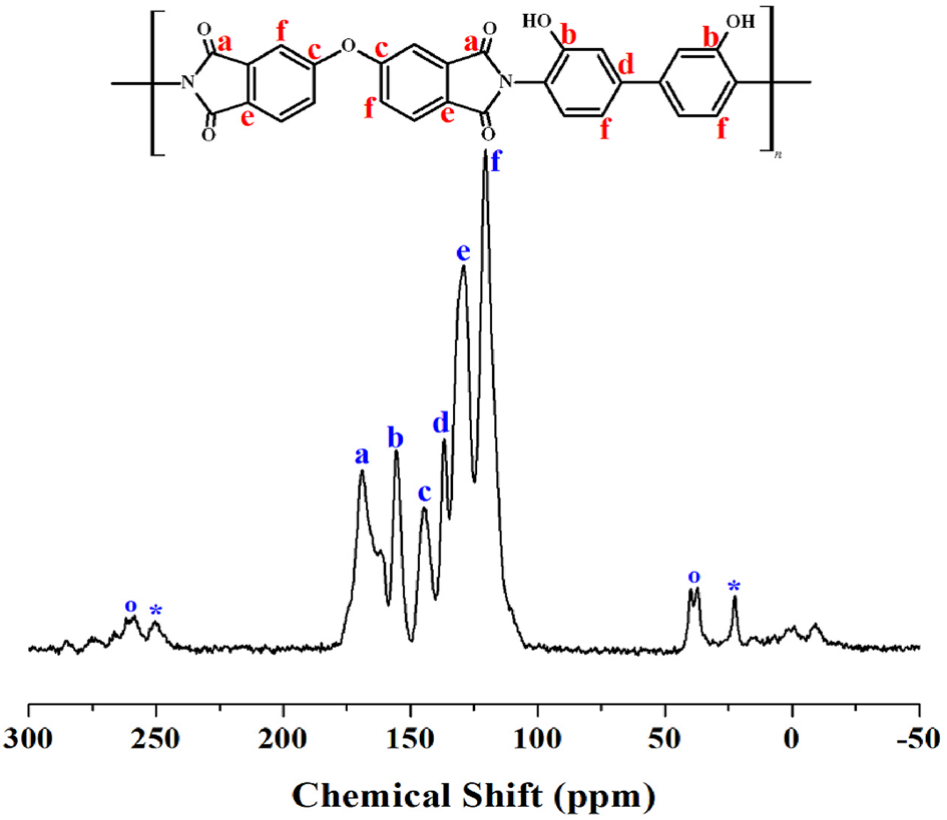
^13^C-MAS NMR spectrum of PI.

Solid-state ^13^C NMR spectroscopy is a technique used to determine the structure of solid-state organic compounds by analyzing the NMR of carbon-13 (^13^C) nuclei. A chemical shift scale is used to interpret the solid-state ^13^C NMR spectrum. In this study, the chemical shifts of the ^13^C nuclei were calibrated to 0 ppm using the tetramethylsilane standard peak at 38.3 ppm at a temperature of 25 °C. In Fig. 3, NMR peaks corresponding to the carbons of imide and phenol were observed at 169.05 (**a**) and 155.54 (**b**) ppm, respectively. The peaks for phenyl carbons were observed at 144.48 (**c**), 136.71 (**d**), 129.42 (**e**), and 120.60 (**f**) ppm, with spinning sidebands indicated by open circles and asterisks^22^. The NMR chemical shifts of all carbons were consistent with the expected chemical structures.

### XRD patterns

**Figure 4:**
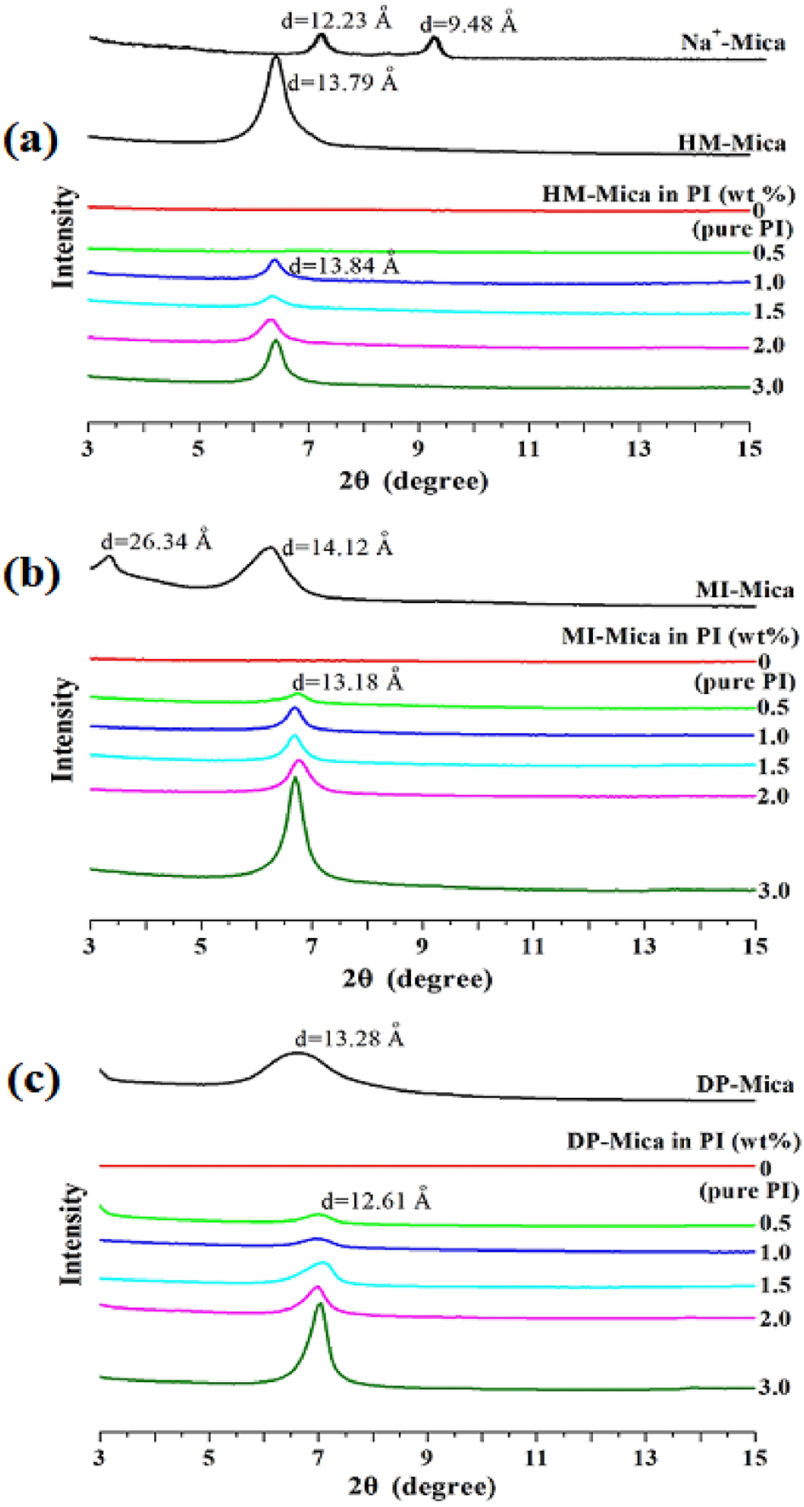
XRD patterns of PI and PI hybrid films with various organo-mica contents. (**a**) HM-Mica, (**b**) MI-Mica, and (**c**) DP-Mica.

In general, an XRD pattern displays the intensity of X-rays scattered by a crystalline material as a function of the diffraction angle (*2θ*). Figure 4 shows the XRD patterns of pristine mica, the organo-micas, and all PI hybrid films with varying organo-mica types and contents plotted against their respective *2θ* values in the range of 3°–15°. The unique and sharp peaks of pristine mica appear at *2θ* = 7.22° (*d* = 12.23 Å) and *2θ* = 9.32° (*d* = 9.48 Å). HM-Mica showed a characteristic peak at *2θ* = 6.40° (*d* = 13.79 Å) (Fig. 4a). The organic material substituted into the clay widened the interlayer distance of the clay layer and facilitated the insertion of polymer chains, thereby enabling the formation of polymer hybrids in which clay is dispersed at the nanoscale level. The XRD patterns of MI-Mica and DP-Mica are similar to those of HM-Mica. The XRD pattern of MI-Mica showed two peaks at *2θ* = 3.35° (*d* = 26.34 Å) and 6.25° (*d* = 14.12 Å) (Fig. 4b). Notably, the *d* = 26.34 Å of MI-Mica is more than twice that of pristine mica (*d* = 12.23 Å). The widening of the interlayer spacing induced by organic mica is believed to facilitate the insertion and dispersion of polymer chains, ultimately improving the physical properties of the hybrid material^15,23^. However, DP-Mica (*d* = 13.28 Å) only slightly increased the interlayer spacing in the PI hybrid film compared with pristine mica (Fig. 4c).

In the hybrid films prepared with HM-Mica, no peak was observed when the amount of organoclay was 0.5 wt%. However, when the amount of organoclay was increased from 1.0 to 3.0 wt%, the intensity of the peak at *2θ* = 6.40° (*d* = 13.84 Å) increased with increasing clay content (Fig. 4a). According to these results, addition of an excessive amount of clay over a certain content may either prevent it from dispersing in the PI matrix or encourage agglomeration^24,25^. The results of MI-Mica and DP-Mica at various contents are similar trends to those of HM-Mica (Figs. 4b,c, respectively). XRD is a simple technique that can be used to evaluate the dispersion of clay and the distance between clay layers dispersed in the hybrid films. In the present study, we consider the XRD results to provide only primary results. Thus, the detailed dispersion of the filler in the polymer matrix was further examined through TEM, the results of which are discussed below^26,27^.

### TEM analysis

While XRD provides information on the crystallographic structure and interlayer spacing of clay minerals, TEM allows researchers to directly visualize individual clay particles in the hybrid film at a much higher resolution. In addition, TEM can be used to quantitatively describe the interlayer structure of the dispersed clay as well as the morphology of the nanoscale dispersion in the PI matrix^28,29^. In all micrographs collected in this study, the black hair-like lines represent 1-nm-thick clay layers, and the gray spaces between these lines represent spaces between layers. For more detailed observations, certain parts of the clay, indicated by arrows, were enlarged.

**Figure 5:**
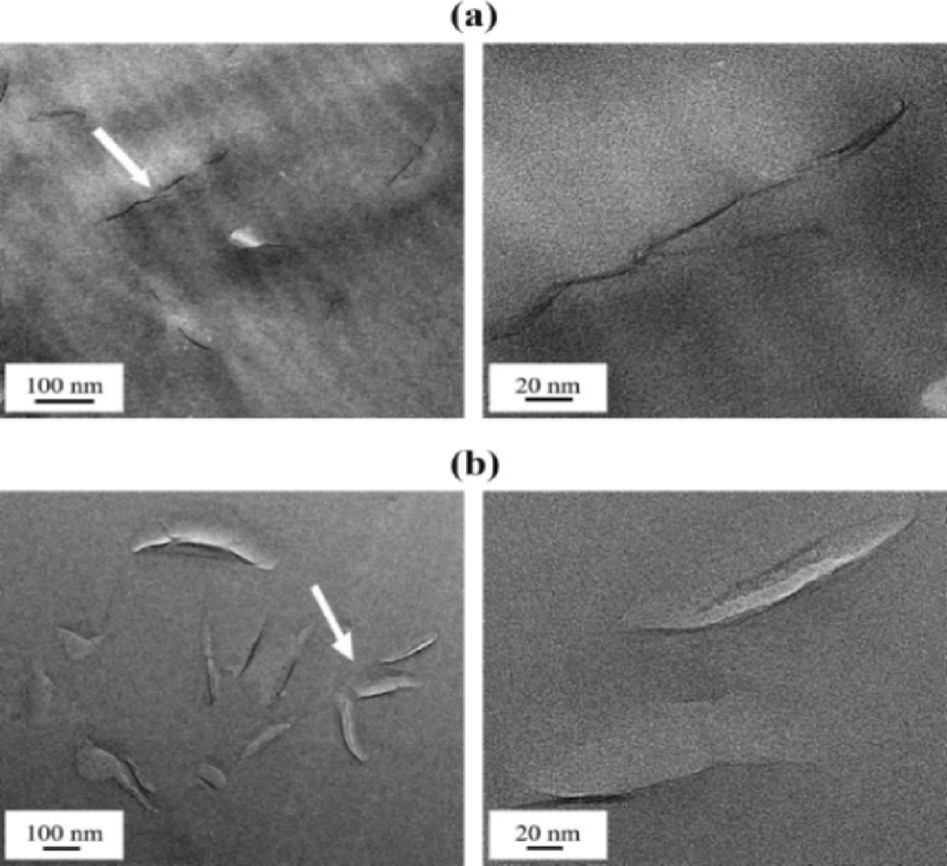
TEM micrographs of PI hybrid film containing (**a**) 1.0 and (**b**) 1.5 wt% HM-Mica.

**Figure 6:**
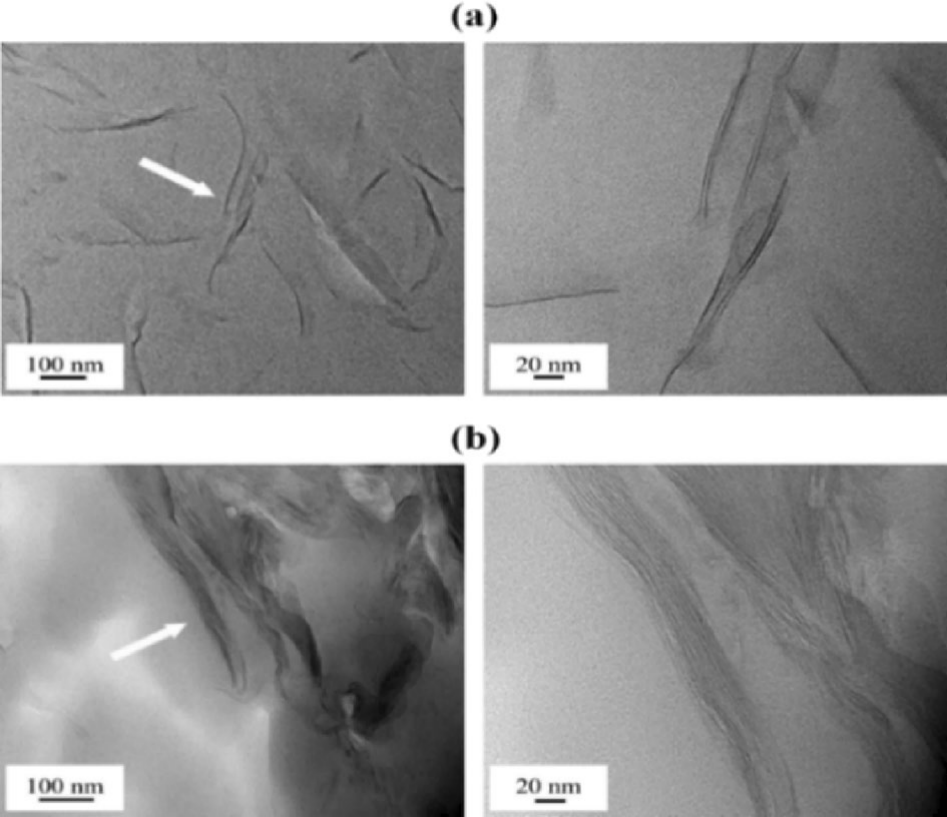
TEM micrographs of PI hybrid film containing (**a**) 1.5 and (**b**) 2.0 wt% MI-Mica.

**Figure 7:**
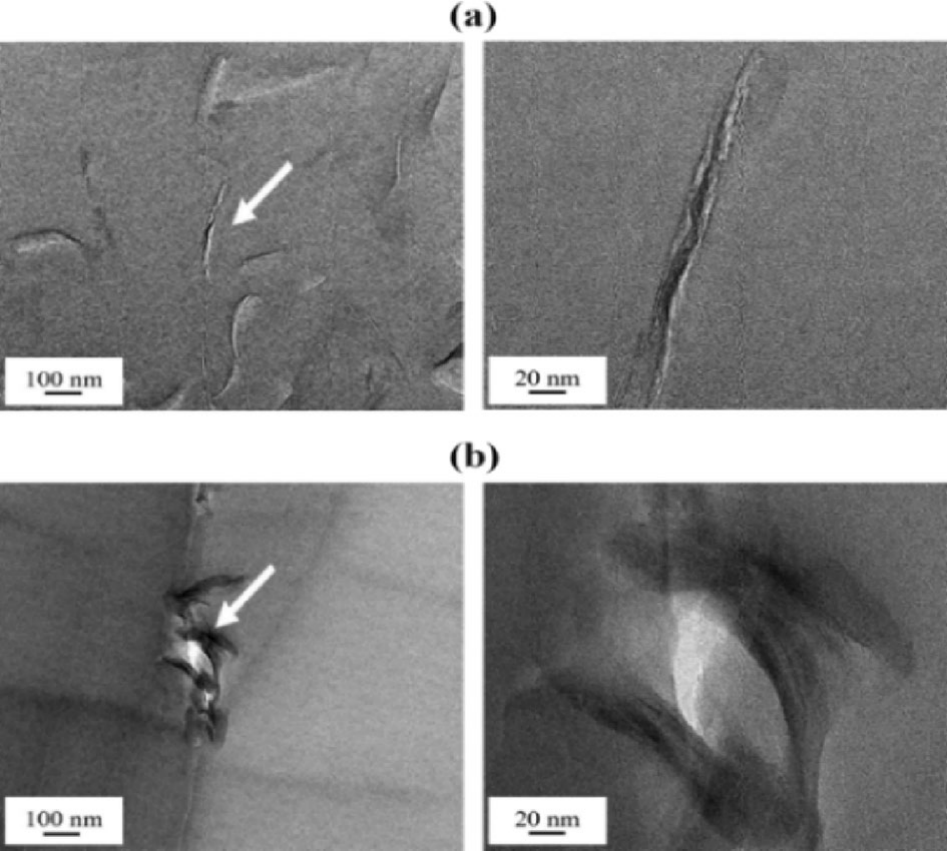
TEM micrographs of PI hybrid film containing (**a**) 1.5 and (**b**) 2.0 wt% DP-Mica.

TEM images of the PI hybrid films containing 1.0 and 1.5 wt% of HM-Mica are shown in Fig. 5. When the HM-Mica content of the hybrid film was 1.0 wt%, the clay was well dispersed in the matrix, with an average thickness of approximately 5 nm, and a straight clay shape was observed, as shown in Fig. 5a. However, when the HM-Mica content was increased to 1.5 wt%, the clay particles aggregated to form a hybrid with an average thickness of approximately 20 nm (Fig. 5b). This result reveals that clay agglomeration occurs in the matrix above a certain critical content. Figure 6 shows TEM micrographs of the PI hybrids prepared with various MI-Mica contents. When 1.5 wt% MI-Mica was dispersed in the matrix, a nanosized hybrid film with only 3–4 layers of aggregated clay was observed (Fig. 6a). However, when the content of MI-Mica was increased from 1.5 to 2.0 wt%, a hybrid film with aggregated clay particles measuring 60–80 nm was formed (Fig. 6b). Similar results were observed for the PI hybrids prepared with various DP-Mica contents. When 1.5 wt% DP-Mica was dispersed in PI, a hybrid film with clay particles measuring approximately 20 nm uniformly dispersed in the matrix was obtained (Fig. 7a). However, when the filler content was increased to 2.0 wt%, the filler aggregated to form particles measuring 30–80 nm (Fig. 7b).

In summary, HM-Mica, MI-Mica, and DP-Mica demonstrated optimal dispersion when added to the PI matrix at contents of 1.0, 1.5, and 1.5 wt%, respectively, and greater aggregation was observed as the clay content increased above these critical contents. Among the three organo-micas investigated, MI-Mica can be expected to induce the best physical properties in the hybrid because it was well dispersed in the matrix at its critical content. The presence of excess clay above the critical content led to agglomeration, which directly affected the thermomechanical properties of the hybrid films, as discussed in the next section.

### Thermal behaviour

**Figure 8:**
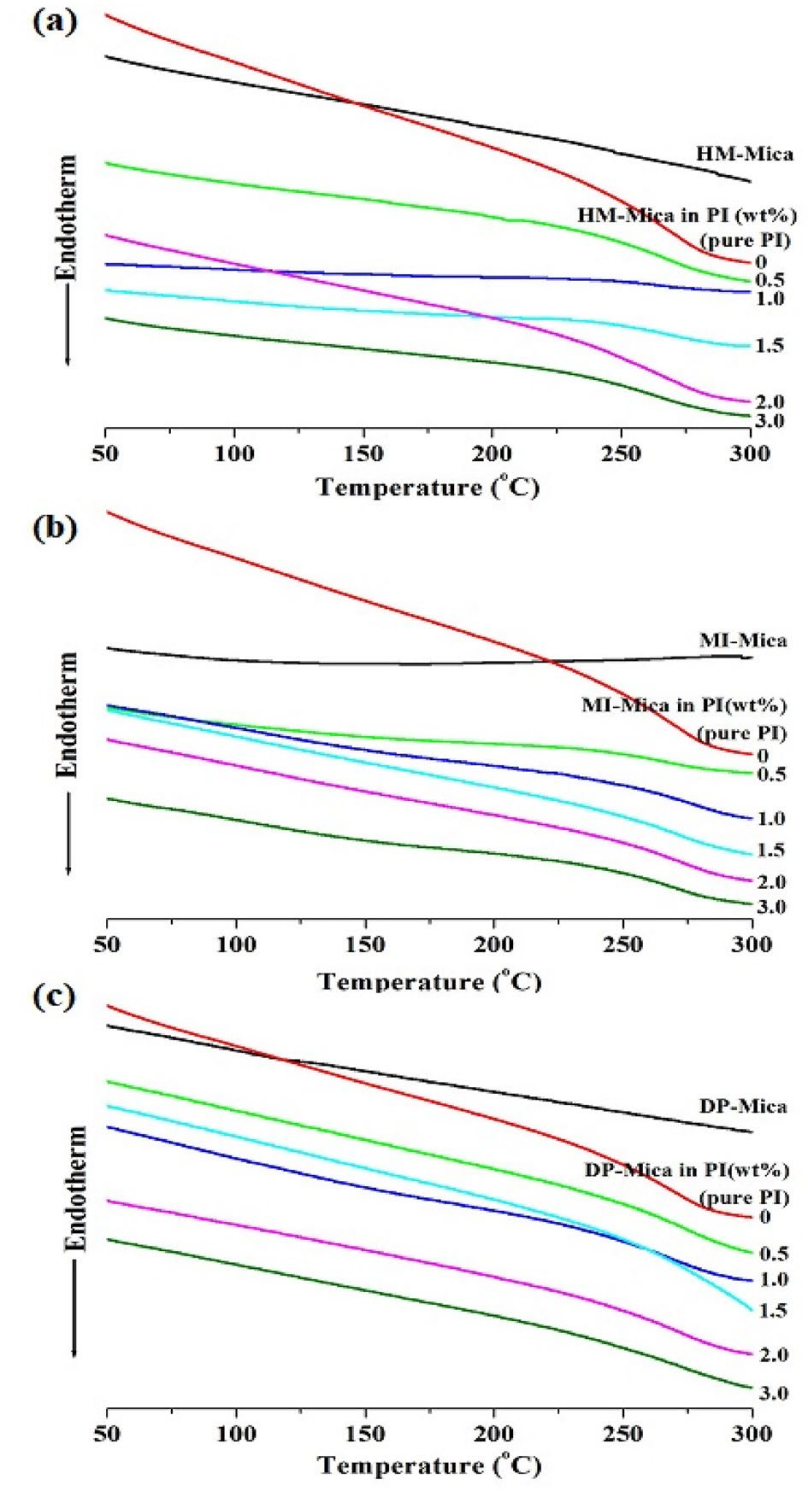
DSC thermograms of PI and PI hybrid films with various organo-mica contents. (**a**) HM-Mica, (**b**) MI-Mica, and (**c**) DP-Mica.

Table 2 summarizes the DSC results of all PI hybrid films, and Fig. 8 illustrates the thermal behaviors of the films. The glass transition temperature (*T*_*g*_) was obtained through secondary heating and was determined as the temperature at the midpoint of the temperature range where the transition occurs in the thermogram. The *T*_*g*_ of the pure PI film was 264 °C. When the HM-Mica content in the PI matrix was increased to 1.0 wt%, *T*_*g*_ increased significantly to 271 °C. This increase in *T*_*g*_ can be explained by two factors, as referenced from previous studies. The first factor is related to the presence of rigid and plate-like mica layers dispersed within the polymer matrix. These clay layers reduce the free volume available for polymer chain movement. As a result, the *T*_*g*_ of the hybrid film increases because of the restricted mobility of the polymer chains^30,31^. The second factor is associated with the hindered segmental motion of the polymer chains intercalated within the interlayer spaces of the clay^32,33^. The presence of the clay restricts the mobility of the polymer chains, leading to an increase in *T*_*g*_. Note that *T*_*g*_ can vary significantly depending on several factors, such as the structure of the monomers, secondary bonds (e.g., hydrogen bonds) present in the polymer chains, chain fluidity, and the presence and concentration of additives^34,35^. When the organo-mica content was further increased to 3.0 wt%, the *T*_*g*_ of the hybrid film decreased to 259 °C. This decrease in *T*_*g*_ can be attributed to the aggregation of the clay particles, which begins to occur above the critical clay content^19,27,36^.

**Table 2:** Thermal properties of the PI hybrid films.

Organoclay in PI (wt%)	HM-Mica				MI-Mica				DP-Mica			
	*T*_*g*_ (°C)	$T_{D}^{ia}$(°C)	$wt_{R}^{600b}$(%)	CTE^c^ (ppm/°C)	*T*_*g*_ (°C)	$T_{D}^{i}$(°C)	$wt_{R}^{600}$ (%)	CTE (ppm/°C)	*T*_*g*_ (°C)	$T_{D}^{i}$(°C)	$wt_{R}^{600}$ (%)	CTE (ppm/°C)
0 (pure PI)	264	258	70	36	264	258	70	36	264	258	70	36
0.5	264	298	71	29	266	280	71	31	264	261	73	35
1.0	271	303	74	28	272	287	73	28	266	265	73	33
1.5	264	287	71	29	279	307	73	27	271	280	74	27
2.0	261	252	70	30	274	276	73	28	269	263	71	33
3.0	259	253	72	30	266	275	72	30	266	259	70	33

^a^Initial decomposition temperature at 2% weight loss.

^b^Weight residue at 600 °C.

^c^Coefficient of thermal expansion for the second heating at 50–200 °C.

Overall, the incorporation of organo-micas into the PI hybrid films had a significant impact on their thermal behavior, as indicated by the changes in *T*_*g*_ with varying clay content. The results suggest that an optimal clay content should be chosen to achieve the desired properties in the hybrid film by maximizing the benefits of mica dispersion and avoiding excessive aggregation. Similar to the case of the hybrid films prepared with HM-Mica, the *T*_*g*_ of the hybrid films prepared with MI-Mica and DP-Mica increased when the organo-mica content was low but decreased when the organo-mica content was higher than the critical content. For example, when the MI-Mica content was increased to 1.5 wt%, *T*_*g*_ increased to 279 °C. However, when the content of the organo-mica was increased to 3.0 wt%, *T*_*g*_ decreased to 266 °C. The *T*_g_ of the hybrid film prepared DP-Mica was highest at 271 °C when the filler content was 1.5 wt% but decreased to 266 °C when the filler content was 3.0 wt% (Table 2). The TEM results in Figs. 5, 6, 7 support this finding by revealing the presence of clay aggregation.

**Figure 9:**
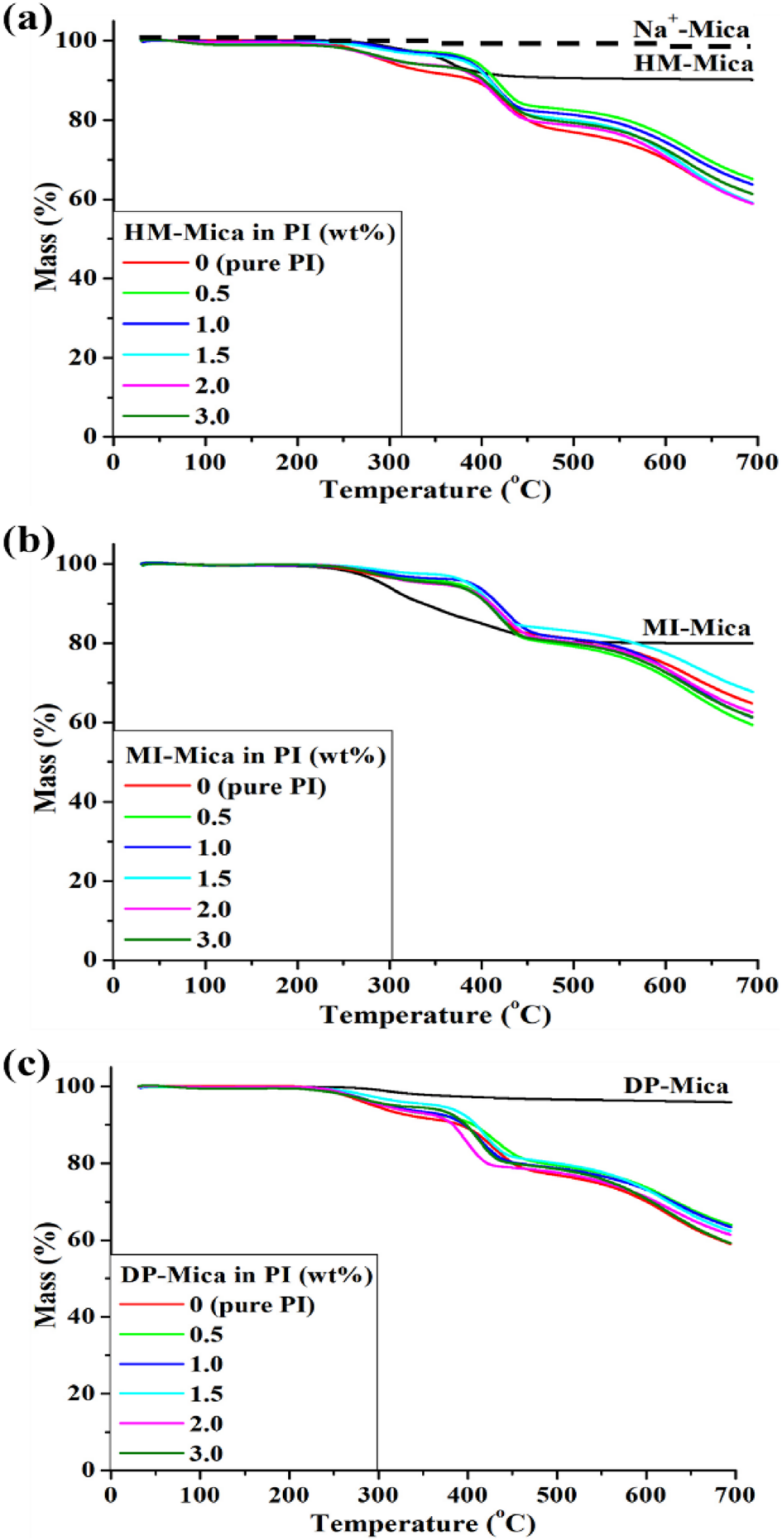
TGA thermograms of PI and PI hybrid films with various organo-mica contents. (**a**) HM-Mica, (**b**) MI-Mica, and (**c**) DP-Mica.

The initial decomposition temperature ($T_{D}^{i}$)is a parameter that refers to the thermal stability of a substance at an early stage of heating. It represents the temperature at which the decomposition process begins and provides valuable information on the mechanism and behavior of the substance during decomposition. The TGA results of the pure PI and all PI hybrid films are shown in Fig. 9, and their thermal stabilities are summarized in Table 2.

**Scheme 2:**
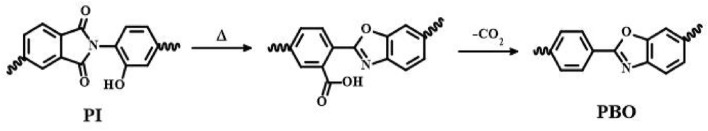
Thermal rearrangement of PBO from PI by heat treatment.

Mica, which is pristine clay, did not decompose even at 700 °C and showed excellent thermal stability. However, all the organically modified micas showed an initial weight loss at approximately 250 °C. This result can be explained by the low thermal stability of the alkyl groups substituted in the organo-micas. Coincidentally, the $T_{D}^{i}$ of pure PI was approximately 250 °C, similar to that of organo-mica. The lower *T*_*D*_^*i*^ of pure PI compared with those of the PI hybrids can be attributed to the presence of –OH substituents with low thermostability in the main chain of PI, as shown in Fig. 8. According to Fig. 9, several stages of thermal decomposition were observed upon heating in TGA of PI and PI hybrid containing DHB monomers. This is because PI with -OH group undergoes thermal rearrangement (TR) at high temperature to change to polybenzoxazole (PBO)^37,38^. Most PIs containing -OH groups are completely converted to PBO by TR through heat treatment above about 450 °C or higher. Scheme 2 shows the process in which PI containing an -OH group becomes PBO through TR. Therefore, the degradation of perfectly formed PI and PBO was observed around 350 and 550 °C, respectively^39^.

When small amounts of organo-mica were added to the PI matrix, notable changes in $T_{D}^{i}$ were observed. For instance, when up to 1.0 wt% HM-Mica was dispersed in the PI matrix, $T_{D}^{i}$ increased to 303 °C from an initial value of 258 °C. Similarly, when 1.5 wt% MI-Mica or DP-Mica was dispersed in the matrix, $T_{D}^{i}$ values of 307 and 280 °C, respectively, were obtained. These changes in *T*_*D*_^*i*^ can be explained by the plate-shaped mica with excellent thermal stability acting as a barrier that effectively impedes the transfer of heat and reduces the volatilization of the PI components^40–42^. When the mica is evenly dispersed within the PI matrix, it hinders heat transfer, especially at higher temperatures. However, when the content of the organo-mica exceeds the critical amount, $T_{D}^{i}$ starts to decrease regardless of the type and content of the organoclay in the hybrid. For example, when the organoclays were added at concentrations above the critical contents for HM-Mica (1.0 wt%) and MI-Mica and DP-Mica (1.5 wt%), the $T_{D}^{i}$ values gradually decreased as the organo-mica content increased, reaching a minimum value at 3.0 wt% (Table 2). These results suggest that the thermal stability effect of the clay is poorly exerted when it is not uniformly dispersed in the PI matrix and, instead, agglomerates as the clay content exceeds the critical amount. This observation is consistent with the previously observed trends for *T*_*g*_.

The residual weight ($wt_{R}^{600}$) of the pure PI and all PI hybrid films at 600 °C was mostly constant at 70–74%, as shown in Table 2. This finding may be attributed to the excellent heat resistance of mica itself and PI, which persists even after nearly all organo-alkyl groups with low thermal stability substituted in the clay are decomposed at high temperatures.

When a pure polymer is heated, it tends to relax and expand in the direction perpendicular to its main chain. However, when plate-shaped clay particles are dispersed evenly in the polymer matrix, the deformation caused by heating becomes is impeded because the clay particles, owing to their hardness and strength, can effectively hinder heat transfer and suppress the lateral thermal expansion of the polymer^43–45^. The high thermal stability of the clay particles allows them to withstand heat without significant expansion or degradation. When thermally stable fillers, such as clay, are evenly dispersed in the polymer matrix, the hybrid material can exhibit low-thermal-expansion characteristics, which is desirable in various applications, particularly in electronic and high-temperature materials.

**Figure 10:**
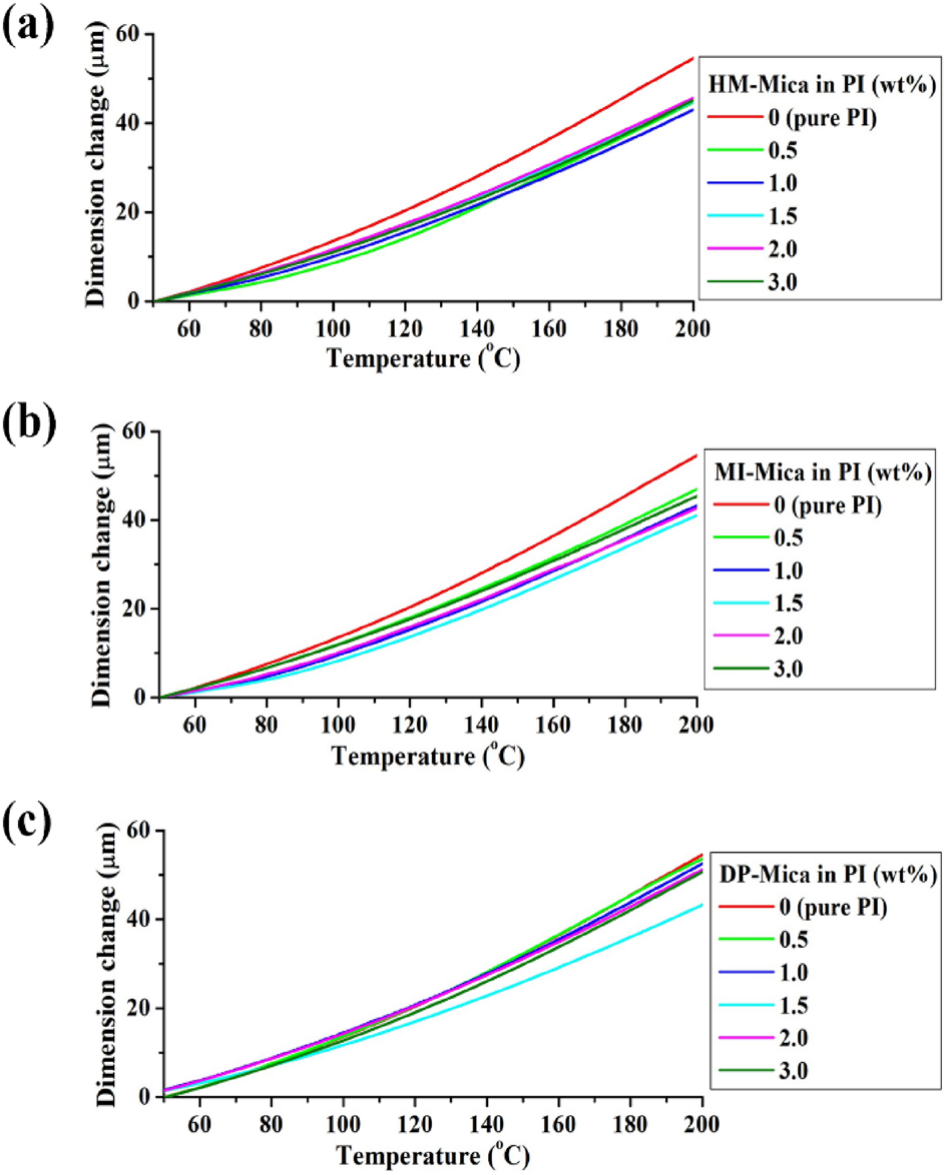
TMA thermograms of PI and PI hybrid films with various organo-mica contents. (**a**) HM-Mica, (**b**) MI-Mica, and (**c**) DP-Mica.

The Fig. 10 shows the TMA results of the PI hybrid films at temperatures between 50 °C and 200 °C. The results obtained from the TMA analysis are also summarized in Table 2. The initial CTE of pure PI was 36 ppm/°C. However, when 1.0 wt% HM-Mica was dispersed in the PI matrix, the CTE of the hybrid significantly decreased to 28 ppm/°C. Interestingly, even when higher contents of HM-Mica (up to 3.0 wt%) were dispersed in the matrix, the CTE of the hybrid film remained nearly constant at 30 ppm/°C. The CTEs of the MI-Mica and DP-Mica hybrids decreased to a low value of 27 ppm/°C when the organ-mica content reached the critical amount of 1.5 wt%. However, beyond this critical content, the CTE began to increase once more as the organo-mica content further increased. These results indicate that the addition of organo-mica can significantly influence the CTE of the PI hybrid films. A small amount of HM-Mica (1.0 wt%) caused a notable decrease in CTE, but further increases in organo-mica content did not significantly affect the CTE. The type of organo-mica (MI-Mica and DP-Mica) also played a role in determining the CTE behavior, with the MI-Mica and DP-Mica hybrids exhibiting the lowest CTE values at the critical content of 1.5 wt%.

The results so far indicate superior efficiency compared to the other two organo-mica hybrids in terms of thermal properties even when MI-Mica is added at the same concentration. Specifically, incorporation of 1.5 wt% MI-Mica (i.e., the critical MI-Mica concentration) into the hybrid film yielded optimal results. The enhanced thermal properties of the hybrid can be attributed to the presence of organo-alkyl groups attached to the surface of the mica particles. In the case of MI-Mica, these organo-alkyl groups consist of long alkyl chains, particularly hexadecyl (C_16_-) groups. These long alkyl chains contribute to the widening of the interlayer spacing of the mica clay, which facilitates the insertion of polymer chains^46–48^. This phenomenon is supported by the *d* values obtained from the XRD analysis shown in Fig. 4. A larger *d* value indicates a larger interlayer spacing, which, in turn, promotes the dispersion of polymer chains among the clay particles. This improved dispersion allows for a more uniform distribution of the polymer chains within the clay matrix, leading to enhanced thermal properties. The positive impact of MI-Mica on the thermal properties of the hybrid film is likely to be reflected in its mechanical properties, as will be discussed in the next section.

### Mechanical tensile properties

The mechanical properties of all PI hybrid films were examined by measuring their ultimate tensile strength, initial modulus, and elongation at break (EB). Table 3 summarizes the mechanical properties of the hybrid films.

**Table 3:** Mechanical properties of the PI hybrid films.

Organoclay in PI (wt%)	HM-Mica			MI-Mica			DP-Mica		
	Ult. Str.^a^ (MPa)	Ini. Mod.^b^ (GPa)	EB^c^ (%)	Ult. Str. (MPa)	Ini. Mod. (GPa)	EB (%)	Ult. Str. (MPa)	Ini. Mod. (GPa)	EB (%)
0 (pure PI)	74	1.20	4	74	1.20	4	74	1.20	4
0.5	85	1.23	5	95	1.23	4	87	1.26	7
1.0	109	1.35	6	101	1.40	7	94	1.37	7
1.5	105	1.14	8	131	1.66	6	118	1.46	8
2.0	76	1.04	8	84	1.11	7	106	1.30	8
3.0	75	0.89	8	83	1.05	6	96	1.16	8

^a^Ultimate strength.

^b^Initial modulus.

^c^Elongation at break.

The mechanical properties of the hybrid films, similar to their thermal properties, reached maximum values at a specific critical content but decreased above this critical content. For example, when up to 1.0 wt% HM-Mica was dispersed in the pure PI film, the ultimate strength and initial modulus of the hybrid material increased from 74 to 109 MPa and from 1.20 to 1.35 GPa, respectively. However, when 3 wt% HM-Mica was used, the ultimate strength and initial modulus of the hybrid film decreased to 75 MPa and 0.89 GPa, respectively. That is, the mechanical properties of the hybrid films increased up to the critical organo-mica content but decreased above this content. This decrease in ultimate tensile strength and initial modulus can be explained by the agglomeration of clay particles, which occurs mainly with the addition of clay above the critical content because the excess clay acts as a defect in the hybrid film.

Similar results were observed in the hybrid films prepared with MI-Mica and DP-Mica. That is, the ultimate strength and initial modulus of the hybrid films reached maximum values at the respective critical contents of these fillers but decreased above these contents. When 1.5 wt% MI-Mica was dispersed in the pure PI film, the ultimate strength and initial modulus of the hybrid film were 131 MPa and 1.66 GPa, respectively, corresponding to increases of approximately 80% and 40% compared with those of pure PI. When DP-Mica was used, the tensile strength and initial modulus of the hybrid film increased by 60% and 20%, respectively, compared with those of pure PI. In addition, the critical contents of MI-Mica and DP-Mica appeared to be higher than that of HM-Mica, and the mechanical properties of the hybrid film prepared with MI-Mica were better than those of hybrid films prepared with HM-Mica and DP-Mica. This finding may be attributed to the ability of the long hexadecyl groups substituted in MI-Mica to widen the gap between the clay layers and facilitate the insertion of polymer chains, thereby promoting the dispersion and orientation of the clay in the polymer matrix^49–51^. These results reveal that mica substituted with organic compounds of a specific structure have a significant effect on the tensile properties of the hybrid films. The EB of pure PI was 4%, but that of the hybrid films containing clay was almost constant in the range of 5–8% regardless of the type and content of organo-mica used.

Based on the results obtained thus far, the longer the organic compound substituted on the clay surface, the more effectively the distance between the clay layers was widened. When the hybrids were prepared, the critical contents of the organo-micas that brought about maximum effects also differed depending on the degree of compatibility, dispersibility, and interactions of the polymer chain with the structure of the organic compounds substituted on the clay surface. Therefore, structural differences among the organic materials substituted on the clay exerted distinct effects on the thermomechanical properties of the resultant hybrid films.

### Optical transparencies

Light transmittance is used to evaluate the optical properties of a film and is closely related to the clay content. Specifically, as the amount of clay dispersed in the matrix increases, the light transmittance of the hybrid gradually decreases because the plate-shaped clay does not transmit light. The optical transparency of the PI hybrid films was evaluated in terms of the cut-off wavelength (λ_o_), which refers to the initially transmitted wavelength, transmittance at 500 nm (500 nm^trans^) in the visible-light region, and YI^52^.

**Figure 11:**
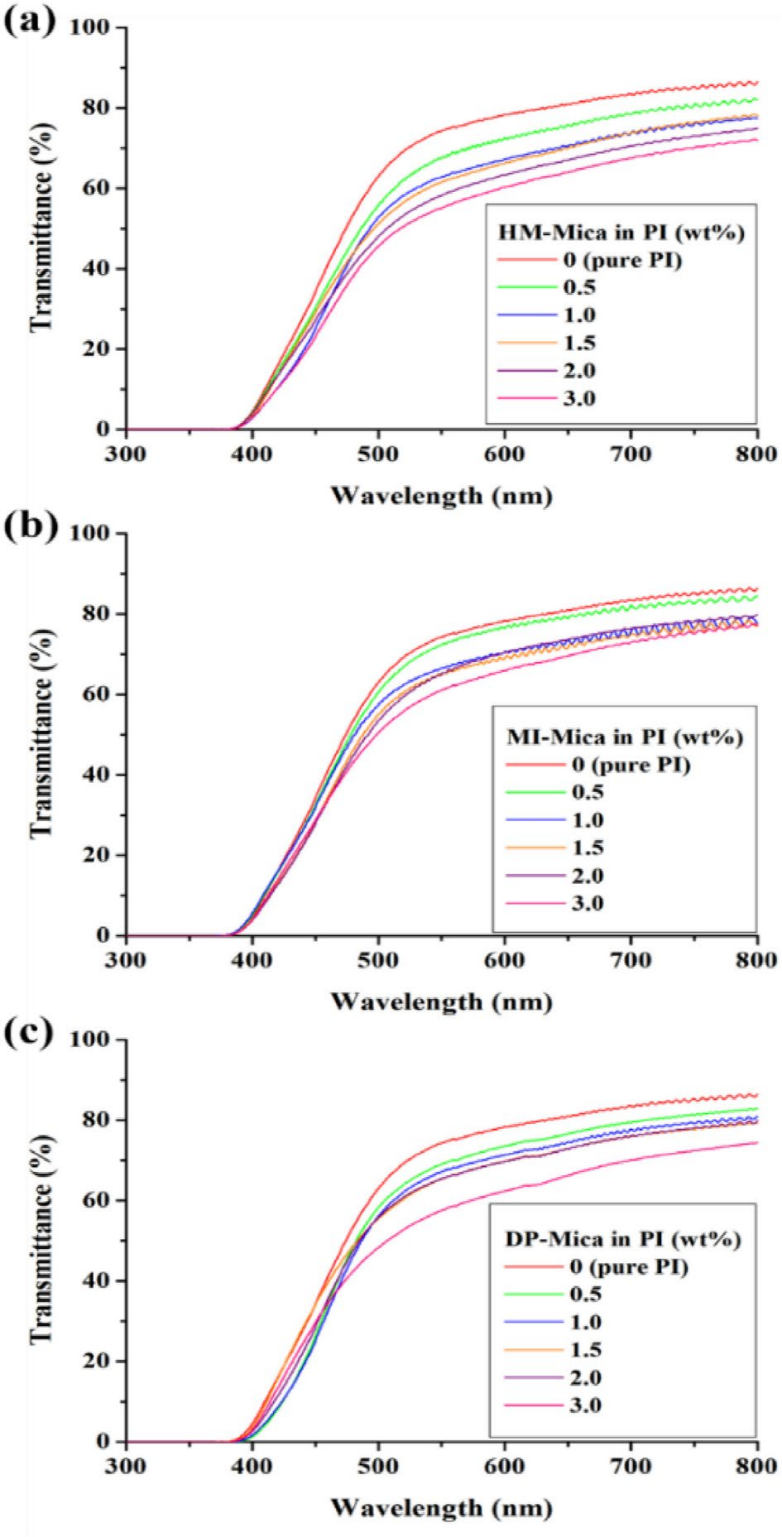
UV–vis. transmittances of PI and PI hybrid films with various organo-mica contents. (**a**) HM-Mica, (**b**) MI-Mica, and (**c**) DP-Mica.

The UV–vis. spectra of the hybrid films are shown in Fig. 11, and a summary of the optical properties of these films is given in Table 4. The thickness of the hybrid films was adjusted to 19–22 μm to enable the comparison of their optical properties under identical conditions. The λ_o_ of the PI hybrid films showed a consistent value of 370 nm regardless of the type and content of organo-mica used. This finding means that the λ_o_ of all synthesized PI hybrids is less than 400 nm and that all films transmit light before the visible-light region. By contrast, the 500 nm^trans^ of the hybrid films decreased steadily as the clay content increased from 0 to 3.0 wt%. For example, when the contents of HM-Mica, MI-Mica, and DP-Mica in the PI matrix were increased from 0 to 3.0 wt%, the 500 nm^trans^ values of the hybrid films decreased from 62 to 45%, 50%, and 48%, respectively. The reduction in 500 nm^trans^ may be attributed to the dispersion of the plate-shaped mica clay in the PI matrix, which hinders light transmission in the visible region. Therefore, as the clay content increased, the transmittance of the hybrid films gradually decreased. The YI of the pure PI film was 46, but all hybrid films nearly consistently showed YI values of 45–48 regardless of the type and content of organo-mica used. This result reveals that the filler dispersed in the matrix hardly affected YI. In most previous cases, as the amount of clay in the hybrid increased, YI also increased owing to clay aggregation, which contradicts the findings in the present study. The TEM results (Figs. 5, 6, 7) suggest that if an excessive amount of clay is added to the PI matrix, the YI of the hybrid increases because of the aggregation of the clay. However, in this study, the degree of aggregation was mild; thus, we can assume that YI is not greatly affected by the presence of the filler.

**Table 4:** Optical properties of the PI hybrid films.

Organoclay in PI (wt%)	HM-Mica				MI-Mica				DP-Mica			
	Thickness^a^ (μm)	$\lambda_{0}^{b}$(nm)	500nm^trans^ (%)	YI^c^	Thickness (μm)	λ_0_ (nm)	500 nm^trans^ (%)	YI	Thickness (μm)	λ_0_ (nm)	500 nm^trans^ (%)	YI
0 (pure PI)	21	370	62	46	21	370	62	46	21	370	62	46
0.5	20	370	55	47	21	370	60	46	20	370	58	46
1.0	20	370	52	48	21	370	57	45	20	370	56	47
1.5	22	370	51	47	21	370	54	46	19	370	55	45
2.0	21	370	48	48	22	370	53	48	19	370	55	45
3.0	20	370	45	48	21	370	50	47	20	370	48	47

^a^Film thickness.

^b^Cutoff wavelength.

^c^Yellowness index.

**Figure 12:**
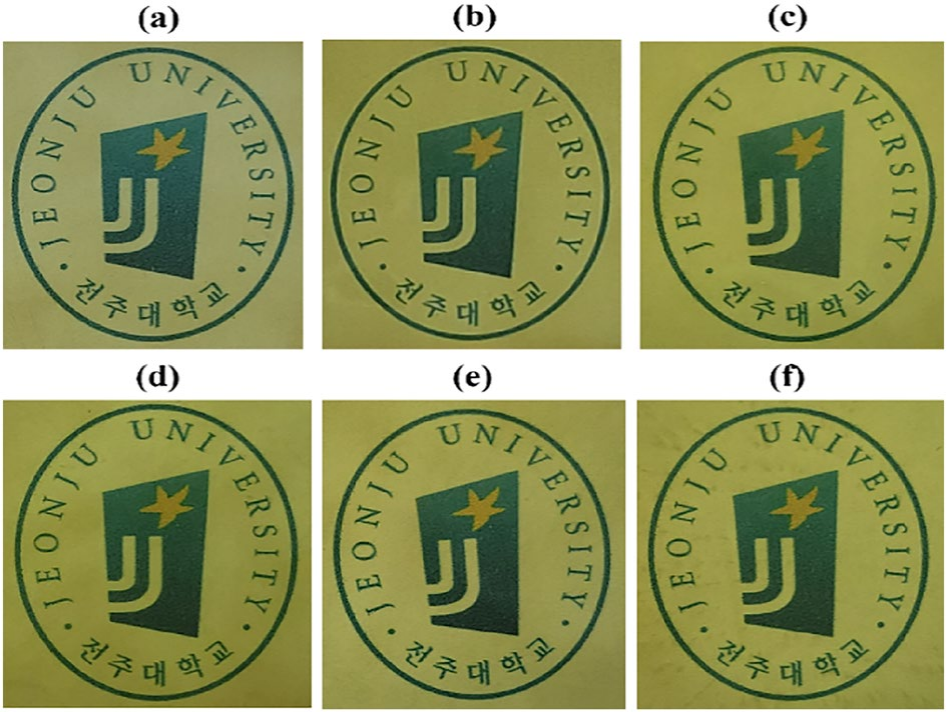
Photographs of PI hybrid films with various organo-mica contents. (**a**) 0 (pure PI), (**b**) 0.5, (**c**) 1.0, (**d**) 1.5, (**e**) 2.0, and (**f**) 3.0 wt% HM-Mica.

The optical properties of the prepared films can be observed from the photographs of the actual PI hybrid films shown in Fig. 12. The hybrid films prepared with HM-Mica was light brown in color and transparent regardless of their HM-Mica content. Also, the logo placed beneath the films was easy to read. The optical properties of the hybrid films prepared with MI-Mica and DP-Mica were similar to those of the film prepared with HM-Mica regardless of the filler content (Supplementary Figures S1 and S2).

## Conclusion

PAA was synthesized using dianhydride ODPA and diamine DHB, and PI hybrid films were prepared by dispersing three types of organo-micas in PAA under various heat-treatment conditions using a solution intercalation method. The thermomechanical properties, morphology, and optical transparency of PI hybrid films containing different filler types and contents were measured and compared. Some of the dispersed organo-mica aggregated in the films, but XRD and TEM confirmed that most of it was dispersed at the nanoscale level. When the organo-mica added to the PI matrix reached a critical content, the physical properties of the hybrid films showed maximum values. However, when the amount of organo-mica added to the matrix exceeded a critical content, the physical properties of the hybrid films deteriorated owing to the agglomeration of the mica. Among the three fillers used to prepare the PI hybrid films, MI-Mica induced the best physical properties, and its critical content was higher than that of HM-Mica.

According to the results obtained in our experiments, organic compounds with longer chains substituted on the clay surface of organo-mica can more effectively widen the distance between the clay layers and the dispersibility, compatibility, and interaction of the polymer chains with these organic compounds depend on the organo-mica’s structure. We confirmed that the critical content of organo-mica that brought about the best effects in the hybrid materials differed depending on the degree of dispersion of the clay and that the structure of the filler greatly affected the thermomechanical properties and morphology of the hybrid films.

### Supplementary Information

Supplementary Information.

## Data Availability

The datasets used and/or analysed during the current study available from the corresponding author on reasonable request.
