# Supplementary material for: Effects of various types of organo-mica on the physical properties of polyimide nanocomposites
Source: Sci Rep. 2024 Jan 5;14:655. doi: 10.1038/s41598-023-51064-6 (PMC10770344; doi:10.1038/s41598-023-51064-6)
Supplement: Supplementary file 1 — Supplementary Information. [file 41598_2023_51064_MOESM1_ESM.docx]

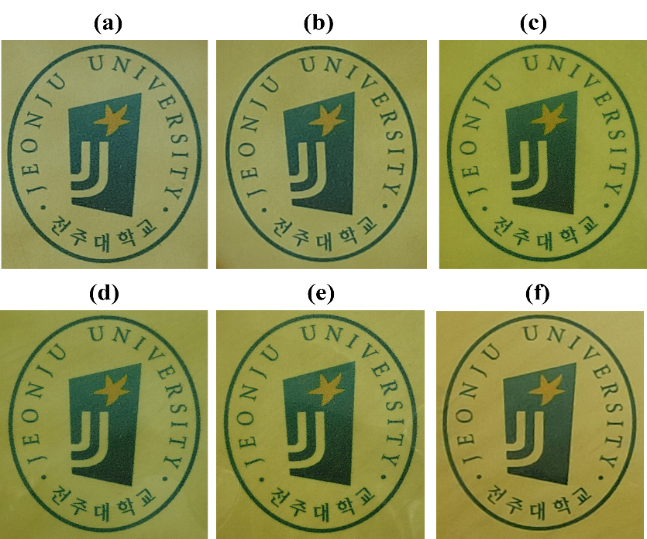


**Supplementary-Figure S1.** Photographs of PI hybrid films with filler contents.

(a) 0 (pure PI), (b) 0.5, (c) 1.0, (d) 1.5, (e) 2.0, and (f) 3.0 wt% MI-Mica.


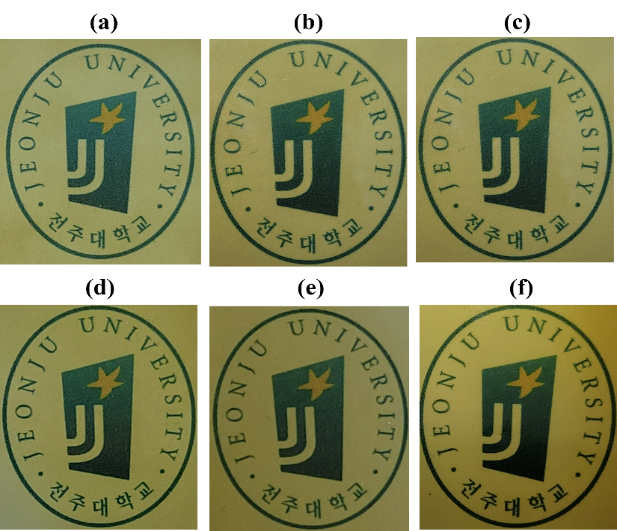


**Supplementary-Figure S2.** Photographs of PI hybrid films with filler contents.

(a) 0 (pure PI), (b) 0.5, (c) 1.0, (d) 1.5, (e) 2.0, and (f) 3.0 wt% DP-Mica.
